# Evaluation of In-Vitro Activity of Ceftaroline Against Methicillin-Resistant Staphylococcus aureus Clinical Isolates

**DOI:** 10.7759/cureus.49859

**Published:** 2023-12-03

**Authors:** Ankita Roy, Nirmala Poddar, Kumudini Panigrahi, Basanti Pathi, Subham Ravi Nayak, Roshni Dandapat, Dipti Pattnaik, Ashok K Praharaj, A. Raj Kumar Patro

**Affiliations:** 1 Microbiology, Kalinga Institute of Medical Sciences, Bhubaneswar, IND

**Keywords:** real-time pcr, e-test, meca gene, ceftaroline, mrsa, amr

## Abstract

Methicillin-resistant *Staphylococcus aureus* (MRSA) is one of the major causes of hospital and community-acquired infections. Fewer drugs, such as vancomycin, teicoplanin, and daptomycin, are effective against it, but they come with high toxicity. Fifth-generation cephalosporins like ceftaroline and second-generation cefuroxime are effective against MRSA. Limited studies are available on ceftaroline resistance in the literature. This study was undertaken to determine ceftaroline resistance in MRSA in a tertiary care hospital in Eastern India. A cross-sectional, hospital-based study was carried out with MRSA isolates obtained from various clinical samples of patients. Identification of the isolates to the species level was performed by an automated Vitek system, and selected samples were genotypically confirmed by detecting the *mecA* gene via real-time PCR. Out of a total of 334 *Staphylococcus aureus* isolates examined in this study, the prevalence of MRSA was seen in 59.3% (198/334), and methicillin-sensitive *Staphylococcus aureus* was in 40.7% (136/334). Of the total 198 MRSA isolates, ceftaroline intermediate MRSA was seen in 8.6% (17/198), and ceftaroline sensitive MRSA was in 91.4% (181/198), respectively. Among the 17 ceftaroline intermediate MRSA isolates, 88.2% (15/17) showed a minimum inhibitory concentration (MIC) of 2 µg/ml, and 11.8% (2/17) showed an MIC of 3 µg/ml. All the remaining 91.4% (181/198) isolates were sensitive to ceftaroline and showed an MIC ≤1 µg/ml. Real-time PCR confirmed the presence of the *mecA* gene in MRSA isolates. In this present study, not a single isolate was resistant to ceftaroline, suggesting that it, being a safer drug, can be used in place of glycopeptides such as vancomycin or teicoplanin and linezolid, where resistance has already been detected. The rational use of ceftaroline could be useful in clinical settings, and further studies will confirm the findings.

## Introduction

*Staphylococcus aureus *(*S. aureus*) is a major human pathogen that causes both community-acquired and hospital-acquired infections. It is a leading cause of nosocomial infection, especially in patients with devices, catheter-related infections, infective endocarditis, skin and soft tissue infections, and in immunocompromised conditions [1]. Treatment of *S. aureus* becomes challenging due to the emergence of drug-resistant strains such as methicillin-resistant *S. aureus *(MRSA), which are capable of spreading resistance to other antibiotics, such as penicillin and aminoglycosides, through horizontal gene transfer. MRSA has long been a pathogen in hospital-acquired infections (HA-MRSA), but now it is also reported in community settings (CA-MRSA). Vancomycin is the drug of choice and sometimes the last resort for the treatment of MRSA [2]. However, reduced susceptibility and the emergence of resistance to vancomycin in MRSA cases are on the rise. New drugs are being evaluated for their efficacy against MRSA. Ceftaroline is an advanced-generation cephalosporin approved by the United States Food and Drug Administration for the treatment of adults with acute bacterial skin and soft tissue infections, including MRSA [3].

The production of an altered penicillin-binding protein (PBP2a), encoded by the *mecA* gene by a *Staphylococcus* clinical isolate, results in resistance to methicillin. Ceftaroline binds to penicillin-binding proteins, inhibiting cell wall synthesis, and has a high affinity for PBP 2a, which is associated with methicillin resistance [4]. Ceftaroline is a newer agent with little information on its susceptibility pattern. It can be considered an effective alternative treatment for MRSA isolates because it has demonstrated promising potency and is much safer than vancomycin. The ability of ceftaroline to bind to PBP 2a, an MRSA-specific protein, makes it unique among cephalosporins. Ceftaroline is consistently active against multidrug-resistant *Streptococcus pneumoniae*, *S. aureus*, and a few Gram-negative organisms [5].

Ceftaroline is a newer agent, and information on its susceptibility pattern is limited. Very few studies have been undertaken to evaluate the susceptibility of MRSA to ceftaroline in different countries, including India, and there is limited data about the susceptibility pattern [6]. The objective of our study was to evaluate the susceptibility pattern of ceftaroline (fifth-generation cephalosporin) against MRSA.

## Materials and methods

This cross-sectional study was conducted in a tertiary care hospital from November 2020 through May 2022 after obtaining ethical clearance from the institute ethics committee (KIIT/KIMS/IEC/405/2020). During the study period, clinical isolates were obtained from various clinical samples of patients attending the outpatient department or admitted to various clinical departments. Data collected included demographic details, brief clinical history, details of the clinical diagnosis with any existing or past co-morbidities, and the antibiotic susceptibility pattern of patients with Staphylococcus aureus infection.

Bacterial isolates and antimicrobial susceptibility testing

Identification of the clinical isolates to the species level was performed by the automated VITEK 2 system (bioMérieux). Phenotypic identification of MRSA isolates was performed by cefoxitin disk diffusion using a cefoxitin disk (30 μg). Susceptibility of the isolates to ceftaroline was carried out using E-test MIC test strips (Biomerieux, France) containing a concentration gradient range of CPT (0.002-32 μg/ml). Breakpoints for the interpretation of ceftaroline minimum inhibitory concentration (MIC) were carried out according to CLSI (M100, 31st edition guideline: Sensitive ≤ 1 μg/ml, Intermediate 2-4 μg/ml, Resistant ≥ 8 μg/ml). ATCC 29213 *S. aureus* was used as a control strain for MIC detection [6]. Selected isolates were subjected to genotypic confirmation by detecting the *mecA* gene using a Taqman probe-based real-time PCR (TRUPCR MRSA detection kit, version 3.0, by 3B Blackbio Biotech, India).

Phenotypic detection of MRSA by cefoxitin disk diffusion test

These *S. aureus* strains were screened for MRSA using a cefoxitin disc according to the CLSI guideline, 2021 (M100, 31st edition) using a 30 μg disk. All isolates were subjected to a cefoxitin disk diffusion test. A bacterial suspension of the clinical isolate was made (0.5 McFarland standard) and a lawn culture was performed on MHA plates. A cefoxitin disc was applied within 15 minutes after inoculation, and plates were incubated at 37°C for 24 hours.

Molecular detection of *mecA* gene

DNA extraction from select isolates was done using a commercial spin column as per the manufacturer’s instructions (TRUPCR nucleic acid extraction kit, India). The purity and concentration of genomic DNA were measured using 1 μl of the eluted DNA in a Nanodrop Spectrophotometer absorbance at 260 and 280 nm (Multiskan Sky, Thermo Scientific, USA). The eluted DNA was amplified for the *mecA* gene target (for confirmation of MRSA) using probe-based Real-time PCR (Quantstudio 5, Applied Biosystems, USA). Each run constituted an internal control (in ROX) for sample adequacy, *S. aureus* (in FAM), and the *mecA* gene target (in VIC). The real-time PCR conditions used were Initial denaturation at 94°C for 10 minutes, denaturation for 15 seconds, annealing at 60°C for 45 seconds, and extension at 72°C for 15 seconds for 40 reaction PCR cycles. Results were interpreted from the Ct value of the amplified product (as per kit cutoff value).

## Results

Out of 334 *S. aureus* isolates, MRSA was phenotypically isolated in 198 samples. One hundred ninety-eight non-duplicate MRSA isolates from various clinical samples were included in the study. The prevalence of MRSA among the 334 *S. aureus* isolates examined was 59.3% (198/334), and methicillin-sensitive *S. aureus* (MSSA) was 40.7% (136/334). Out of the 198 MRSA isolates, 8.6% (17/198) were ceftaroline intermediate, and ceftaroline sensitive MRSA was seen in 91.4% (181/198), respectively. The prevalence of MRSA was higher in males at 55.6% compared to females at 44.4%. The prevalence of ceftaroline intermediate isolates was seen in males at 53% compared to 47% in females. Among ceftaroline intermediate isolates, the majority of cases were seen in the age group 21-40 years (35.3%), followed by the 41-60 years and 61-90 years age groups, respectively (29.4%). Of the 198 MRSA-detected patients, the spectrum of clinical presentation was most commonly seen with urinary tract infections in 19.7% (39/198), skin and soft tissue infections in 11.7% (23/198), with the most common co-morbid condition being diabetes in 12.1% (24/198), hypertension in 16.2% (32/198), and chronic kidney disease in 9.0% (18/198) of cases.

**Figure 1:**
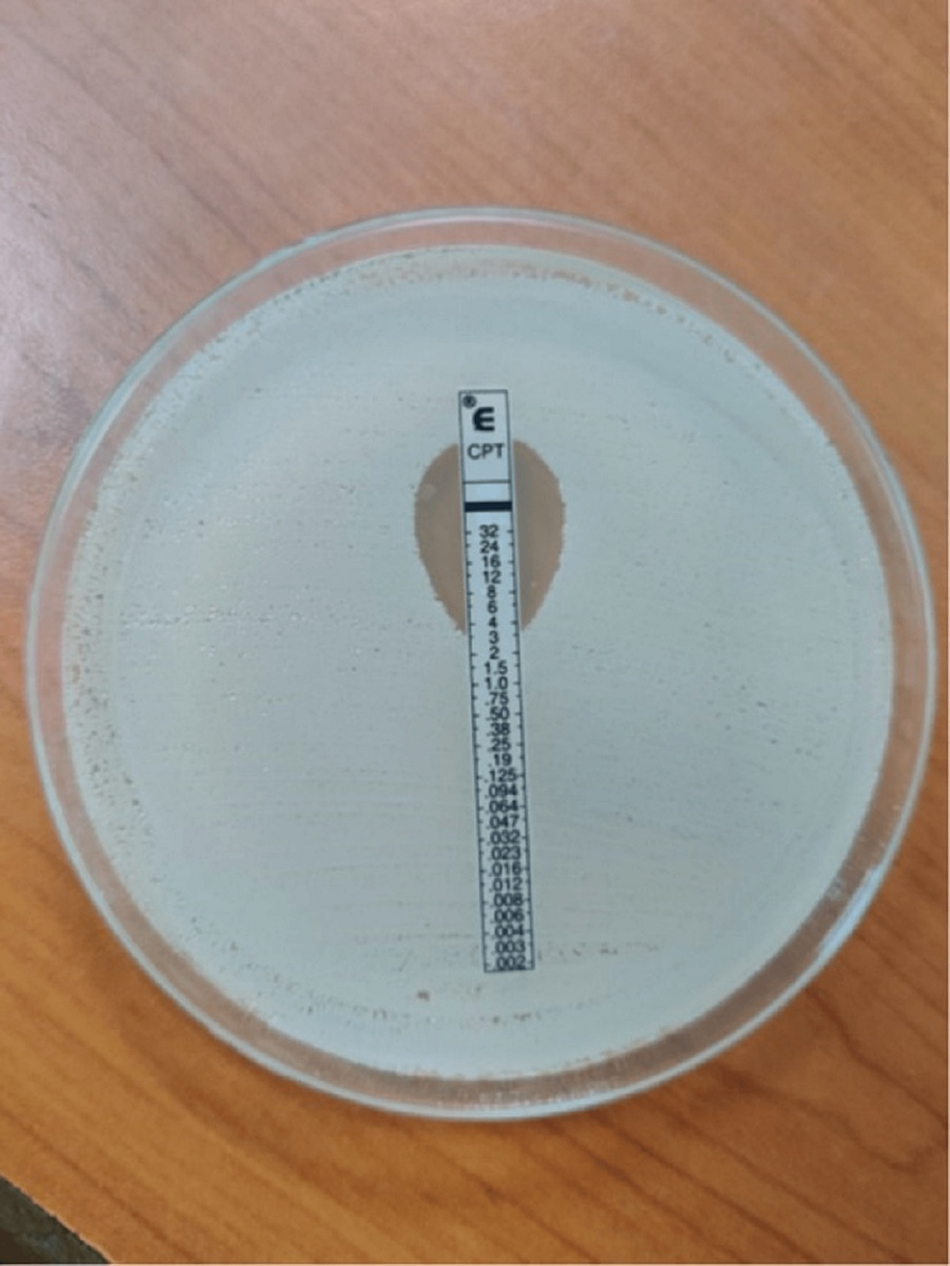
Determination of MIC of ceftaroline by E test Ceftaroline showing MIC = 3 μg/ml (intermediate 2-4 μg/mL) MIC: minimum inhibitory concentration

**Figure 2:**
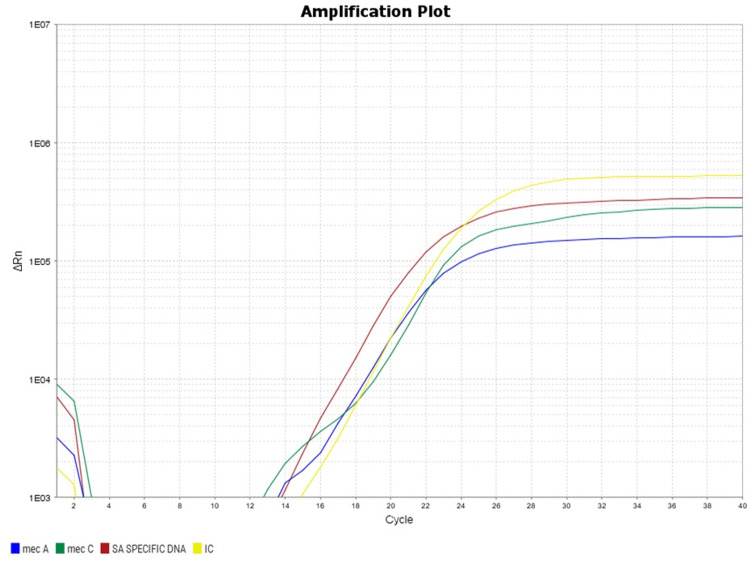
Amplification plot showing molecular detection of mecA gene amplification by real-time PCR of representative samples Real-time PCR amplification of representative sample showing amplification of *mecA* gene. Yellow: internal control, Red: *Staphylococcus aureus*, Green: *mecC* target, and Blue: *mecA* gene target (Applied Biosystems QuantStudio 5 Real-Time PCR System)

Out of the 198 MRSA isolates, 8.6% (17/198) showed intermediate susceptibility, of which 88.2% (15/17) showed an MIC of 2 µg/ml and 11.8% (2/17) showed an MIC of 3 µg/ml. All the remaining isolates, 181 (91.4%), were sensitive to ceftaroline and showed an MIC ≤ 1µg/ml. The MIC50 of isolates was 0.25 µg/ml and MIC90 was 0.38 µg/ml by E-test against MRSA, as shown in Table 1. MIC determination of ceftaroline by E-test and ceftaroline showing MIC = 3 μg/ml (Intermediate 2-4 μg/mL) is shown in Figure 1. Molecular detection of MRSA for the presence of the mecA gene in selected isolates was confirmed by Real-time PCR using a Taqman probe-based method, as shown in Figure 2. MRSA clinical isolates were detected in various specimens, with the most clinical isolates being 33% (65/198) in urine samples, followed by 18% (35/198) in blood specimens. The prevalence of HA-MRSA was 62.1%, and CA-MRSA was 37.9%. The distribution of MRSA clinical isolates in other different clinical samples is shown in Table 2. However, no MRSA clinical isolates were detected in throat swabs, bile, semen, bronchoalveolar lavage, and vitreous fluid tested in this study. Among the 17 ceftaroline intermediate isolates, 70.6% (12/17) were seen in urine samples, followed by 11.8% (2/17) in blood samples, as shown in Table 3. Of the 17 ceftaroline intermediate isolates, 64.7% (11/17) were HA-MRSA. Ceftaroline intermediate MRSA isolates were maximally sensitive to vancomycin (100%) and tigecycline (88.2%), followed by linezolid (76.5%), teicoplanin (64.7%), gentamicin (23.6%), and clindamycin (23.6%), as shown in Table 4.

**Table 1:** Determination of MIC of ceftaroline by E-test against MRSA MIC: minimum inhibitory concentration, MRSA: methicillin‑resistant *Staphylococcus aureus*

Ceftaroline E-Test	
MIC Values (in μg)	No. of Isolates
0.032	2
0.064	5
0.125	19
0.25	82
0.50	55
1	18
2	15
3	2
4	0

**Table 2:** Distribution of MRSA isolates in different clinical samples (N=198) MRSA: methicillin‑resistant *Staphylococcus aureus*

Specimen	MRSA, n(%)
Blood	35(17.7)
Urine	65(32.9)
Pus	32(16.1)
Endotracheal secretions	14(7.0)
Vaginal swab	12(6.0)
Pleural fluid	3(1.5)
Ascitic fluid	4(2.0)
Sputum	6(3.0)
Tissue	7(3.6)
Swab	5(2.6)
Cerebrospinal fluid	4(2.0)
CVP tip	1(0. 5)
Body fluid (aspiration)	1(0.5)
Wound swab	8(4.0)
Blood dialysis line	1(0.5)

**Table 3:** Distribution of ceftaroline intermediate MRSA in different clinical samples (N=17) MRSA: methicillin-resistant *Staphylococcus aureus*

Specimen	Ceftaroline Intermediate MRSA, n (%)
Blood	2 (11.8%)
Urine	12 (70.6%)
Ascitic fluid	1 (5.9%)
Sputum	2 (11.8%)

**Table 4:** Antibiotic susceptibility pattern of ceftaroline intermediate MRSA isolates S = Sensitive, I = Intermediate, R = Resistant MRSA: methicillin-resistant *Staphylococcus aureus*

Antibiotics	S, n (%)	I, n (%)	R, n (%)
Gentamicin (GEN)	4(23.6)	3(16.7)	10(58.9)
Ciprofloxacin (CIP)	1(5.9)	0	16(94.1)
Clindamycin (CD)	4(23.6)	0	13(76.5)
Tigecyline (TGC)	15(88.2)	0	2(11.8)
Teicoplanin (TEI)	11(64.7)	1(5.9)	5(29.4)
Linezolid (LZ)	13(76.4)	0	4(23.6)
Vancomycin (VAN)	17(100%)	0	0

## Discussion

*S. aureus* causes a range of clinical diseases and is a leading cause of both hospital and community-acquired infections. Over the years, *S. aureus* has become resistant to antibiotics, making the treatment of MRSA infections a significant challenge for clinicians. The emergence of multi-drug resistant MRSA isolates complicates the treatment of MRSA infections [7]. With the rise of antimicrobial resistance among *S. aureus* isolates, particularly MRSA strains, newer drugs are needed to treat these life-threatening infections both in community and healthcare settings. Ceftaroline, a newer fifth-generation cephalosporin, has been approved as a treatment option for severe MRSA infection [7]. Its side effects are much lesser compared to the usual treatment regimen, i.e., glycopeptides like vancomycin and teicoplanin. Very few studies are available in world literature and in India regarding ceftaroline's susceptibility to MRSA [8-10]. Hence, this study was undertaken to find out the in vitro susceptibility to ceftaroline. Various clinical samples like blood, urine, pus/wound swab, high vaginal swab, CSF, respiratory secretions like sputum, ET secretions/tips, bronchoalveolar lavage, throat swab, and body fluids such as pleural, ascitic, peritoneal fluids were tested for the isolation of *S. aureus*. In this study, ceftaroline intermediate isolates were seen in males at 53% compared to 47% in females. Among ceftaroline intermediates, the maximum number of cases were between the age group 21-40 years (35.3%) followed by the 41-60 years and 61-90 years age groups, respectively (29.4%). Among MRSA, the prevalence was higher in males (55.6%) than females (44.4%), consistent with other studies; Humphreys et al. reported a prevalence of 52.2% in males among MRSA from a survey of 67,412 patients from 590 US facilities [11]. The prevalence of MRSA in this study was 59.3%, consistent with other studies from different regions [12, 13]. Anupurba et al. reported a prevalence of MRSA in a tertiary care hospital to be 54.9% in different clinical specimens. Shantala et al. reported a prevalence of MRSA at 54.8%, while Tiwari et al. examined 783 isolates of *S. aureus* and reported 38.4% MRSA cases from a hospital in northern India [14-16]. The ANSORP study, a multinational surveillance study in different Asian countries, reported HA-MRSA prevalence of 86.5% in Sri Lanka, 77.6% in South Korea, 74.1% in Vietnam, 57% in Thailand, 65% in Taiwan, and 56.8% in Hong Kong, with a lower prevalence of 22.6% in cases [17]. According to Kuehnert et al., a national survey in the USA reported 43.2% MRSA cases [18]. Varied prevalence of MRSA has been reported from different regions of the world [19,20].

In a report by Tsering et al., MRSA among carrier screening samples was seen in 61.9% in throat swabs, 56.5% in sputum, 50% in blood samples, 27% in pus samples, and 45.8% in urine samples [21]. Among different clinical specimens, in our study, MRSA isolation was most commonly detected in urine samples (32.9%), followed by blood samples in 17.7% of cases and pus in 16.1% of cases. Our data are consistent with the Indian Network for Surveillance of Antimicrobial Resistance (INSAR) study, in which the detection of MRSA isolates was predominant in urine at 52%, followed by 48% in blood, 41% in respiratory samples, and 40% in pus samples [22]. Colonization of the skin by MRSA is a key reason for the increasing number of isolates. This leads to the chances of invasion with the use of invasive approaches as seen in intensive care units [23].

In our study, the maximum number of MRSA isolates was from inpatients at 63.1% (125/198) compared to outpatients at 36.9% (73/198). This is similar to findings reported by Lohan et al., who also observed the majority of MRSA isolates among inpatients (75.3%) in comparison to outpatients (24.7%), which could be attributed to the presence of MRSA in the hospital environment [24]. In the present study, the majority of MRSA were sensitive to other antibiotics such as vancomycin (100%), tigecycline (97.4%), linezolid (95%), and teicoplanin (85%), similar to the INSAR study [22]. No MRSA isolate was found resistant to ceftaroline in this study. Gaikwad et al. and Sreedharan et al. [25,26] have reported similar findings. However, a study by Lee et al. reported ceftaroline-resistant MRSA due to clone-specific polymorphism in the PBP2a region [27].

Ceftaroline-intermediate MRSA isolates were sensitive to vancomycin (100%), tigecycline (88.2%) followed by linezolid (76.4%), teicoplanin (64.7%), gentamicin (23.52%), and clindamycin (23.5%), which was similar to other studies [28]. In our study, we did not find any isolate to be completely resistant to ceftaroline, which may be due to the absence of its use in India, as the drug was not introduced when the study was carried out. In the Assessing Worldwide Antimicrobial Resistance Evaluation (AWARE) program, potent in vitro activity of ceftaroline against MRSA was reported [29]. This study emphasizes the need for the use of ceftaroline and points to its use as a strong treatment option for MRSA.

The study has a limitation in that we did not carry out sequencing, as no MRSA strain was fully resistant to ceftaroline. Further, it will not affect the broad findings in terms of the prevalence, further validated by molecular detection of the *mecA* gene in clinical isolates. From our study, we found 17 cases of intermediate susceptibility to ceftaroline that could be amenable to higher doses of treatment, and we did not detect any fully resistant strain of ceftaroline. This could be due to the restricted use of this drug in India and therefore resistance to this drug has not been detected so far. The prevalence of MRSA is constantly changing with the emergence of different resistance strains. The MRSA superbug has evolved virulence mechanisms by encoding genes for resistance. This underscores the need for continuous surveillance and the judicious use of antibiotics [30].

## Conclusions

MRSA causes significant nosocomial as well as community-acquired infections in developing countries. To prevent the spread of the infection in healthcare settings, proper handwashing procedures must be followed. Antimicrobial resistance is inextricably linked to microbial evolution and antibiotic use. MRSA's evolutionary success has been remarkable in this context. In the current study, not a single isolate was resistant to ceftaroline, suggesting that it, being a safer drug, can be used in place of glycopeptides such as vancomycin or teicoplanin, and linezolid, where resistance has already been detected. Further studies are needed to ascertain the findings in different clinical settings.
